# Variable metabolic scaling breaks the law: from ‘Newtonian’ to ‘Darwinian’ approaches

**DOI:** 10.1098/rspb.2022.1605

**Published:** 2022-10-26

**Authors:** Douglas S. Glazier

**Affiliations:** Department of Biology, Juniata College, Huntingdon, PA 16652, USA

**Keywords:** metabolic rate, body size scaling, natural laws, physical constraints, biological regulation, adaptive evolution

## Abstract

Life's size and tempo are intimately linked. The rate of metabolism varies with body mass in remarkably regular ways that can often be described by a simple power function, where the scaling exponent (*b*, slope in a log-linear plot) is typically less than 1. Traditional theory based on physical constraints has assumed that *b* is 2/3 or 3/4, following natural law, but hundreds of studies have documented extensive, systematic variation in *b*. This overwhelming, law-breaking, empirical evidence is causing a paradigm shift in metabolic scaling theory and methodology from ‘Newtonian’ to ‘Darwinian’ approaches. A new wave of studies focuses on the adaptable regulation and evolution of metabolic scaling, as influenced by diverse intrinsic and extrinsic factors, according to multiple context-dependent mechanisms, and within boundary limits set by physical constraints.

## 1. Introduction

Life's size and tempo have been of much interest to scientists because they both relate integrally and pervasively to diverse biological characteristics, including myriad morphological, developmental, physiological, behavioural and ecological traits. For over a century, these two key features of life have also attracted much attention because they are usually inversely related in remarkably regular law-like ways that can be described by simple mathematical formulae. In particular, the relationship between the rate of metabolism (*R*), a commonly used indicator of the ‘pace of life’, and body mass (*M*), a commonly used indicator of ‘body size’, can usually be well described by the simple power function, *R* *=* *aM^b^*, where *a* is the scaling coefficient (antilog of the intercept in a log-linear plot) and *b* is the scaling exponent (slope in a log-linear plot) [1–8]. Accordingly, how fast metabolism proceeds can often be predicted with remarkable accuracy by simply knowing how big an organism is. Also, such predictions can often be extended to the rates and durations of many other biological processes that depend on metabolic energy [4–7,9,10]. In short, the timing of living processes scale with organismal size.

The value and significance of the metabolic scaling exponent *b* have attracted much interest for over the past 150 years. During the late 1800s and early 1900s, many biologists claimed that *b* was universally 2/3 or nearly so, the so-called ‘surface law’, based on simple Euclidean geometry of organismal surfaces across which metabolic resources, wastes and heat are exchanged [11–13]. This belief was initially supported by several intraspecific analyses of ‘metabolic scaling’, especially in various birds and mammals [13]. However, beginning in the 1930s, several analyses of interspecific metabolic scaling caused Max Kleiber and other scientists to claim that a 3/4 exponent was universal or nearly so, the so-called ‘3/4-power law’ or Kleiber's Law [2,3,14,15]. Belief in this law peaked during the 1980s to middle 2000s, as a result of its advocacy by Robert Peters [4], William Calder [5] and Knut Schmidt-Nielsen [6] in three highly cited synthetic books, and the appearance of influential supporting theory based on the geometry and physics of internal resource-supply (RS) networks [10,16–19]. However, hundreds of data analyses showing that metabolic scaling exponents are highly diverse and often significantly different from 3/4 (see §2), and the reporting of many lines of evidence contradicting the assumptions, logic and predictions of theory based on RS networks [8,13,20–31], have together resulted in many biologists abandoning a belief in the 3/4-power law, especially since the middle 2000s. As a result, a paradigm shift in the theory and methodology of metabolic scaling from ‘Newtonian’ to ‘Darwinian’ approaches is occurring, as I review here.

## 2. Variability of metabolic scaling

**Figure 1.**
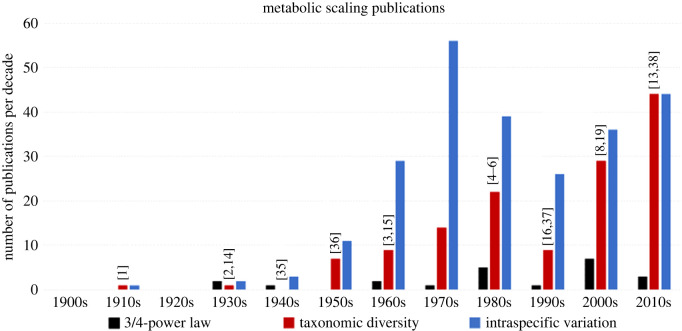
The frequency of publications per decade providing empirical data indicating significant variation of the metabolic scaling exponent (*b*) among species/clades (taxonomic diversity) or within species (intraspecific variation), or apparent uniformity to a 3/4-power law. The decadal timing of some key publications is indicated [1–6,8,13–16,19,35–38], whose locations are unrelated to the coloured bars (see online supplementary information for a list of all publications counted). (Online version in colour.)

The surface law and 3/4-power law, and theory supporting them, have so captivated the minds of many scientists that from the early 1900s to early 2000s, numerous studies showing significant diversity of metabolic scaling have been largely ignored or regarded (especially by theoreticians) as being the result of factors having secondary importance to those causing a presumed primary universal scaling pattern. Ironically, some proponents of the 3/4-power law have suggested that the immense diversity of life has so distracted many biologists that it has inhibited their proclivity to develop general, coarse-grained theory based on universal natural laws [32,33], thus causing them to fail to ‘see the forest for the trees' [33,34]. However, I argue that the opposite has actually occurred: general theory based on a supposed universal law and a single primary deterministic mechanism has inhibited an appreciation of the variability of metabolic scaling and its diverse causes. The history of the theory of metabolic scaling shows that biologists first sought and favoured general explanations based on universal physical laws [13,30] (see also §6). Indeed, it has taken many decades of steadily accumulating studies showing variable metabolic scaling (figure 1) to convince many biologists to abandon general, over-simplistic theory based on a non-existent 3/4-power law. In short, biologists are increasingly ‘seeing the trees for the forest’.

My survey of the literature shows that between 1900 and 2019, 358 studies documented significant variation in intra- or interspecific metabolic scaling exponents for resting or active organisms typically measured under controlled laboratory conditions, as compared to 22 supporting a single universal *b* value (i.e. 3/4) (figure 1). Despite this greater than 16-fold difference and numerous emphatic protests appearing since the 1950s (e.g. [8,13,30,36,39–53]), the 3/4-power law has had a long-lasting tenacious grip on the theory of metabolic scaling. Studies reporting significant intraspecific variation in metabolic scaling exhibited an approximately exponential increase in decadal frequency from the 1910s to 1970s (peaking at 56) and thereafter have continued to appear at high frequency. Although numerous, these studies appear to have had little impact on metabolic scaling theory until recently, apparently because they were often attributed to statistical error (based on relatively narrow body mass ranges within many species [5,15,54,55]; though this is not true for many animals and plants that exhibit indeterminate growth [8,28,46,56,57]), or to factors secondary to those causing the supposed overall 3/4-power scaling pattern, which is said to encompass diverse species exhibiting a very broad range of body sizes [5,10,15,19,32]. However, a sharp increase, especially since the 1990s, in the number of studies showing significant taxonomic variation in metabolic scaling exponents, both among species and clades (peaking at 44 during the 2010s), has made it very difficult to retain a belief in a universal 3/4-power law. Evidence (albeit circumstantial) that a strong belief in a 3/4-power law has actually inhibited studies on the variability of metabolic scaling can be seen in the substantial decline in number of such studies for both intra- and interspecific metabolic scaling during the 1990s and early 2000s, when the synthetic analyses of Peters, Calder, Schmidt-Nielsen and others were frequently cited in the literature [4–6], and resource-transport network (RTN) theory supporting the 3/4-power law had gained prominence [10,16,17,19,32,33]. However, multiple critical reviews in the 2000s and 2010s [8,13,25,30,52,53,58–61] contributed to a more than twofold resurgence of the number of studies documenting significant variation in metabolic scaling, from 34 (1–2 publications every four months) during 1990–1999 to 82 (1–2 publications every two months) during 2010–2019. This trend appears to be continuing into the 2020s (14 publications between January 2020 and November 2021, again between 1 and 2 publications every two months: see online electronic supplementary material).

**Figure 2.**
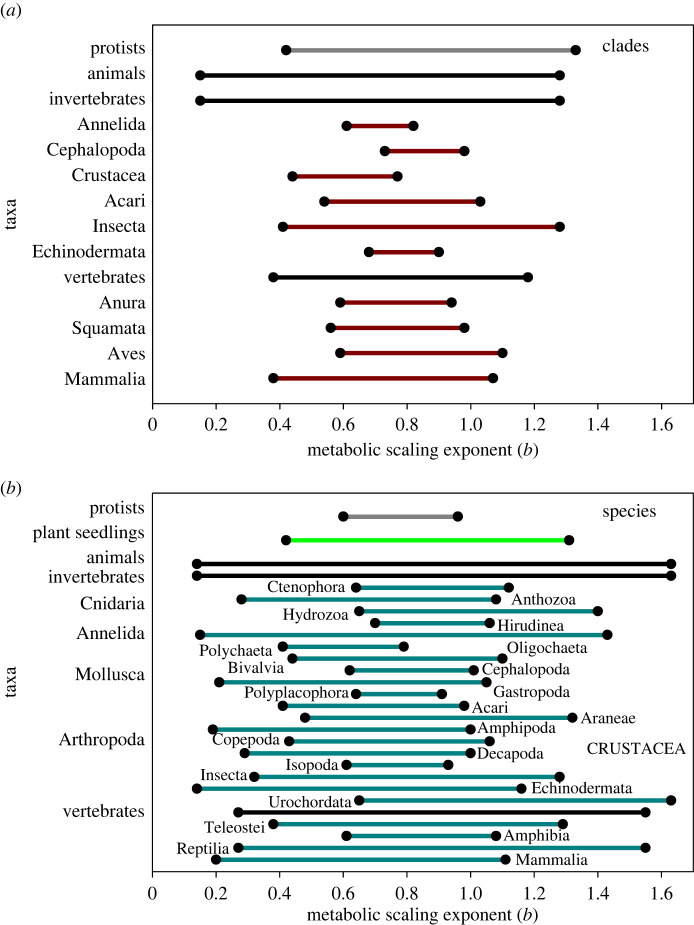
Broad ranges of metabolic scaling exponents (*b*) in diverse taxa of unicellular and multi-cellular organisms (based on ordinary least-squares regressions from sources cited in [4,8,13,25,30,39–42,44,47,56,62–68] (see also electronic supplementary information)). (*a*) Range of interspecific *b* values among clades within various taxa with sufficient data. The grey line refers to unicellular protists, whereas the black lines refer to multi-cellular invertebrates and vertebrates, as whole groups, and the dark red lines refer to taxa within each of these two groups. (*b*) Range of intraspecific *b* values within various taxa with sufficient data. The grey line refers to unicellular protists, whereas the green line refers to multi-cellular plants, the black lines refer to multi-cellular animals, invertebrates and vertebrates, as whole groups, and the blue lines refer to various invertebrate and vertebrate taxa. (Online version in colour.)

The inadequacy of the 3/4-power law is also evidenced by the extremely wide ranges of *b* values (approx. 0.1–1.6 overall, but mostly between 0.5 and 1.0) that have been reported for several taxa of unicellular and multi-cellular organisms (figure 2). The ubiquity of this extensive diversity at various taxonomic levels, including many relationships with wide body mass ranges, and the demonstration of numerous systematic effects, as discussed in §3, suggests that much of this variation is not merely due to statistical or methodological error.

## 3. Diverse intrinsic and extrinsic factors affect metabolic scaling

**Figure 3.**
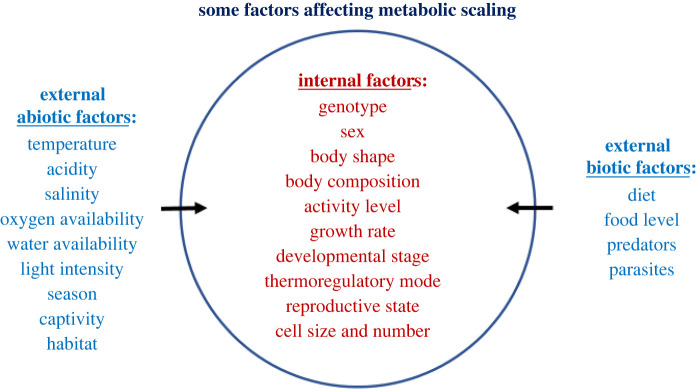
Various internal and external factors causing variation in the metabolic scaling exponent (*b*) (sources cited in text). (Online version in colour.)

Variation in the metabolic scaling exponent (*b*) relates systematically to various intrinsic (biological) and extrinsic (ecological) factors (figure 3), and is not merely statistical ‘noise’ obscuring the recognition of a 3/4-power law. Many of these effects involve major shifts in *b* between approximately 0.5 (or 2/3) and 1.0, as predicted by context-dependent, multi-mechanistic theory [13,25,30,69] (see also §6). Intrinsic effects include significant differences in *b* observed between endothermic versus ectothermic vertebrates [25,38,70,71], active versus resting versus torpid animals [8,25,48,69,72,73], larval versus adult forms [8,28], males versus females [8,74] and various genetic strains [75–77] and cellular modes of growth [44,78]. Extrinsic factors that may affect metabolic scaling include diet, habitat, captivity, ecological lifestyle and various environmental factors, such as temperature, pH, salinity, light intensity, predators, parasites and availability of resources (e.g. oxygen, food and water) [8,46,56,57,62,66,69,79–95]. For example, significantly steeper intraspecific metabolic scaling has been observed for pelagic versus benthic invertebrates [8,46], amphipod populations in spring habitats without versus with fish predators [57], crayfish populations in streams with more monoculture-based agricultural riparian land cover [89], and many ectothermic (especially sedentary) animal and plant species exposed to low versus high ambient temperatures [8,56,69,83,87,90]. In addition, significant differences in interspecific *b* values have been reported for grazing versus folivorous and vertebrate- versus invertebrate-eating mammals [91], arboreal versus terrestrial carnivorans [92], mesic versus desert small mammals [79]; captive versus wild-caught birds [80]; high- versus low-altitude birds [93], temperate versus tropical birds [84]; subtidal versus intertidal gastropods [66], soil- versus wood-feeding termites [94], and crustaceans, fishes, birds and mammals exposed to different ambient temperatures [62,85,95]. Various intrinsic and extrinsic factors may also have interactive effects on *b*. For example, temperature effects have been shown to interact with salinity [40], pH [83], nutrition [96], predation regime [86,88], activity level [87], genotype [97], and mode of thermoregulation [95]. Furthermore, various factors may also cause metabolic scaling to be nonlinear (curvilinear) in log–log space, either within or across species [8,21,28,30,35,63,78,79,82].

## 4. Phenotypic plasticity and evolvability of metabolic scaling

Effects of various intrinsic and extrinsic factors on metabolic scaling may occur via phenotypic plasticity, at least partially driven by biological regulation at the organismal level, or via genotypic evolution driven by natural selection or genetic drift at the population level. Evidence for regulated phenotypic plasticity of the metabolic scaling exponent (*b*) includes dramatic, remarkably regular shifts observed between the allometric scaling (*b* < 1) of resting animals and the isometric or near-isometric scaling (*b* ∼ 1) of torpid or maximally active animals [25,43,69,72,73,98], and other significant shifts in intra- or interspecific *b* in response to individual changes in reproductive state [74,99], social behaviour [13], captivity [80], diet [81], colonial connectedness [100] and exposure to various ambient conditions, especially temperature [8,30,40,69,82,83,85–88,90,95–97]. Evidence for the evolvability of *b* derives from artificial selection experiments [76,101,102], quantitative genetic studies showing significant additive genetic variance and covariance of metabolic rate and body mass [38,76,103], comparative studies of conspecific populations or related species exposed to different natural mortality regimes [8,46,57,88], and phylogenetic studies documenting the timing and direction of evolution of *b* [41,67,104–106].

## 5. Assorted reactions to the demise of the 3/4-power law

There can no longer be any doubt that no universal 3/4-power law exists. However, investigators studying metabolic scaling have reacted to the growing mountain of evidence supporting this inescapable view in diverse ways that I suggest can be approximately classified by using a popular psychological model that posits that people experience five (six) stages of grief after losing a loved one, including ‘denial’, ‘anger’, ‘bargaining’, ‘depression’ and ‘acceptance’ [107], and a recently suggested sixth stage ‘meaning’ [108]. However, according to my modified analogous use of this classification, not everyone may experience all possible psychological stages or do so in a specific order (see [109]), and the loss being considered involves the 3/4-power law, often claimed to be the most accepted natural law in biology [4,16,110–112], an important attribution in a discipline regarded by some as having few or no laws at all, unlike physics [113–115]. I suggest that my approximately comparable classification gives some insight (at least in part) into the ongoing vigorous controversy in the field of metabolic scaling, because various investigators appear to be (or have been) at different stages (singly or in combination) of the process of coping with the empirical ‘death’ of the famous 3/4-power law.

### (a) Denial

Many scientists, especially theoreticians with backgrounds in the physical sciences, continue to believe in a universal or nearly universal 3/4-power law, despite enormous evidence that metabolic scaling is highly diverse across the tree of life (figures 1 and 2), which has continually accumulated ever since the Nobel Prize-winning biologist August Krogh first described variation in the metabolic scaling exponent (*b*) in 1916 [1,51]. This denial (conscious or unconscious) of the demise of the 3/4-power law has occurred, even in the face of numerous recurring studies sharply criticizing the 3/4-power law as being a ‘myth’, ‘unlikely’, ‘doubtful’ or otherwise not being universally applicable that have appeared during the last 65 years [8,13,25,29,30,36,38–53,58–61,64,69,70,72,73,95,105,106]. Nevertheless, a belief in the 3/4-power law has persisted in the minds of many scientists even to the present day [18,19,32,34,112,116,117]. Indeed, during recent years, some theoreticians have continued attempting to explain metabolic scaling simply in terms of a 2/3- or 3/4-power law [18,34,116–121]. A lack of recognition of the demise (inadequacy) of these over-simplistic, supposedly universal laws appears to have resulted from either a lack of knowledge of the extensive literature documenting variable metabolic scaling, or from regarding this variation as being of secondary importance (see also below).

### (b) Anger

During the last two decades, I have witnessed rancorous debates about the existence of the 3/4-power law and theory supporting it at several international scientific conferences. This rancor seems to be driven by conflict between investigators who have different worldviews about how science should be carried out, particularly in biology [8,13,122,123], and who appear to be at different stages of coping with the loss of a universal 3/4-power law.

### (c) Bargaining

Some investigators continue to support the existence of a 3/4-power law by modifying its range of applicability in four major ways. First, some argue that the 3/4-power law applies best to large-scale metabolic scaling relationships encompassing diverse species and taxa with a very broad range of body masses, rather than small-scale relationships within specific species or taxa [5,6,15,19,32,54]. However, this view cannot explain why the mean mass-specific metabolic rate of diverse taxa varies over an unexpectedly limited range (less than 2 orders of magnitude) across approximately 14 orders of magnitude variation in mean body mass [64,124]. Indeed, the mean mass-specific metabolic rate of tiny bacteria is nearly the same as that of large mammals.

Second, some claim that the 3/4-power law applies only to multi-cellular organisms with closed vascular networks, but not unicellular or multi-cellular organisms without closed vascular networks or any circulatory system at all [125]. This view is also problematic because organisms with closed vascular networks (e.g. vertebrate animals and vascular plants) include only a small portion of all species on earth, thus clearly breaking the universality of the law and the theory supporting it. In addition, *b* is not fixed at 3/4 in either vertebrates or vascular plants, but varies considerably between 0.4 or less to 1.2 or more for both intra- and interspecific scaling relationships (figure 2), thus further breaking the law. Diversity of metabolic scaling is pervasive throughout the tree of life.

Third, some posit that a *b* value of 3/4 represents an optimal central tendency for metabolic scaling [15,19,55,126–129] and as such represents a useful ‘rule’ rather than a universal law (e.g. [128], but see [52]). This belief is supported in part by some surveys of metabolic scaling relationships showing that the mean value of *b* is 3/4 or nearly so [4,15,19,127]. However, this view has both empirical and conceptual problems. First, the much-cited classic survey of Robert Peters [4] is biased by an overrepresentation of scaling relationships for vertebrates (72%) and endothermic birds and mammals (44%), which constitute a very small proportion of all living species, as well as in other ways [8]. Second, the frequency histogram presented by Peters shows that 51% of the sampled scaling relationships have *b* values outside 0.7–0.8 [8]. The view that 0.75 is an optimal *b* value thus implies that numerous species with other ‘deviant’ *b* values exhibit suboptimal metabolic scaling. This does not make evolutionary sense because such species should have gone extinct and been replaced by species with the optimal 3/4 value. Third, other surveys have shown that the mean *b* value (± 95% confidence intervals) for intraspecific metabolic scaling relationships is significantly more or less than 3/4 for many major taxa or ecological groups of organisms, such as angiosperms (1.03 ± 0.06, *n* = 9 [130,131]), gastropods (0.67 ± 0.05, *n* = 29 [66]), arachnids (0.854 ± 0.076, *n* = 14 [8]), spiders (0.880 ± 0.036, *n* = 23 [65]), insects (0.830 ± 0.036, *n* = 54 [8]), teleost fishes (0.804 ± 0.030, *n* = 89 [56]; 0.94 ± 0.08, *n* = 55 (16 species) [132]), reptiles (0.670 ± 0.030, *n* = 28 [133]) and pelagic invertebrates (0.947 ± 0.046, *n* = 58 [8,46]), as well as for interspecific relationships including taxonomically heterogeneous groups of animals and plants [8,10,25,124]. In short, *b* varies substantially, not only for individual species values, but also for means (central tendencies) of many large groups of species (approx. 2/3 to 1), often differing significantly from 3/4, which is clearly not a ‘magic’ number [43].

Fourth, some investigators claim that much of the variation in *b* is related to statistical or methodological error [5,6,15,54,55,128], and accordingly, *b* tends to converge toward 3/4 for the most rigorous datasets that have sufficiently large sample sizes and broad body mass ranges, especially over two orders of magnitude [55]. However, close inspection of the graphs (see also [45,134]) used to support this claim actually shows that as the body mass range of a scaling relationship expands, *b* values do not become centred on 0.75, but rather become increasingly confined within the broad boundaries of 0.5 and 1.0. In fact, a greater proportion of *b* values are significantly different from 0.75 for scaling relationships involving larger body mass ranges. For example, in an extensive survey of 642 scaling relationships for 218 animal species, 50.2% were significantly different from 0.75, which increased to 72.7% and 88.5%, respectively, for relationships with body mass ranges at or above 2 or 2.5 orders of magnitude [8,25]. Increasing sample size also causes the frequency of rejection of 3/4-power scaling to increase [8]. Clearly, this is not strong evidence for an optimal value of 3/4.

### (d) Depression

I have talked to colleagues who have given up studying metabolic scaling because they feel that the field has become too acrimonious and divided among largely self-isolated working groups, has failed to make substantive progress during the past few decades and (or) no longer shows promise for developing a general theory. However, as I point out later, general theory need not be monolithic and completely deterministic, but may be multi-faceted and context dependent [13,30]. An ongoing paradigm shift in how metabolic scaling is studied and explained portends many exciting new developments in this important field of study (see §7).

### (e) Acceptance

Since the 1950s, several investigators have accepted the demise (inadequacy) of the 3/4-power law [8,13,30,36–53,59–61,63,69,105,106,125,127], but this recognition has not been widely appreciated by scientists outside the field of metabolic scaling. Based on the existing literature (figure 1), many more investigators in the field of metabolic scaling now reject versus accept a universal 3/4-power law, thus setting the stage for a new wave of research.

### (f) Meaning

Many biologists are now embracing a fundamentally new worldview of metabolic scaling as being highly variable and adaptable, which is revolutionizing the theory, empirical study and practical application of metabolic scaling relationships, as discussed further next.

## 6. Paradigm shift in metabolic scaling from ‘Newtonian’ to ‘Darwinian’ approaches

**Figure 4.**
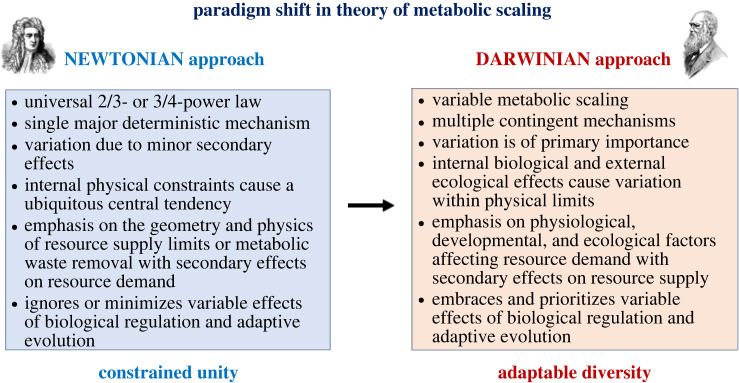
The theory of metabolic scaling has been shifting from ‘Newtonian’ approaches based on physical explanations of a universal law to ‘Darwinian’ approaches based on adaptive regulatory and evolutionary explanations of diverse scaling patterns. Pictures of Isaac Newton and Charles Darwin are freely available from http://clipart-library.com/isaac-newton-cliparts.html and http://clipart-library.com/darwin-cliparts.html. (Online version in colour.)

Biological scaling relationships have been traditionally regarded as physically or developmentally constrained [4,6,10,19,126,135,136], but recently are increasingly being viewed as phenotypically plastic and evolutionarily malleable [37,38,43,53,57,67,77,90,101–106,135–140]. This is particularly true for metabolic scaling. This change in outlook reflects a paradigm shift in general scientific world view and methodology from ‘Newtonian’ approaches emphasizing physically constrained universal laws to ‘Darwinian’ approaches emphasizing adaptable, context-dependent diversity (figure 4 [13]; [141]), as has similarly occurred during the history of other fields of biology. For example, early theories of organic evolution and embryonic development emphasized physical forces acting in deterministic, linearly channelled ways according to natural law (e.g. Lamarckian ‘orthogenesis’ and Haeckel's ‘Biogenetic Law’ or ‘Law of Recapitulation’, where ontogeny recapitulates phylogeny), but were eventually replaced by Darwinian natural selection acting in highly divergent, probabilistic, contextual ways to produce the luxuriantly diverse phylogenies and ontogenies of life that we actually see [142,143].

The ongoing paradigm shift in the theory of metabolic scaling involves five fundamental overlapping changes in focus. Essentially, the primary emphases are shifting from (i) a single universal law to the diversity of metabolic scaling, (ii) a single primary deterministic mechanism to multiple, context-dependent mechanisms, (iii) rigid internal physical constraints to adaptable phenotypic plasticity and genotypic evolution, highly responsive to multiple internal (biological) and external (ecological) causal factors, (iv) centralized to bounded physical constraints and (v) restrictive effects of physically constrained resource supply (RS) and metabolic waste removal across body surfaces and through anatomical transport networks to flexibly regulated and evolvable effects of multiple kinds of resource-demand (RD) that support various vital fitness-related activities.

The shift in focus from an adherence to a single universal law (in particular the 3/4-power law) to embracing the exuberant diversity of metabolic scaling has already been described. Many investigators no longer regard this diversity as being random or secondary to a single primary, physically constrained law, but in itself of primary interest and importance.

Consequently, many investigators have recently formulated several kinds of multi-mechanistic models to explain the diversity of metabolic scaling (reviewed in [13,30]). These models are contextual (situational), as they depend on the biological state of an organism (e.g. its activity level, growth rate, etc.) or its environmental conditions, both biotic and abiotic. For example, the ‘metabolic-level boundaries hypothesis’ (MLBH) posits that the metabolic scaling exponent (*b*) depends on the overall metabolic level of an organism (as estimated by the vertical elevation of a metabolic scaling relationship), which in turn depends on activity level, temperature and other biological and ecological factors in diverse taxa for both intra- and interspecific relationships [8,25,56,65,69,72,73,78,87]. This hypothesis and other kinds of ‘contextual multi-modal theory’ include multiple whole-body size-related mechanisms involving surface and internal transport fluxes of metabolic resources and wastes (including heat), the RD of various biological processes and (or) the proportional masses of tissues with different metabolic demands, among other possible mechanisms at the biochemical and cellular levels, whose relative effects on metabolic scaling appear to vary with context [13,29,30,52,53,78,144].

Each of several possible mechanisms may, by itself, potentially explain some variation in *b*, but clearly not all of the systematic effects of various intrinsic and extrinsic factors that have been observed [13,30]. For example, proponents of RTN theory suggest that simply altering the geometry or physics of transport networks can explain much of the existing diversity of *b* [16,18,126,145]. However, this explanation has several limitations, including that no direct causal relationship between variation in the geometry of RTNs and whole-body metabolic scaling has yet been empirically demonstrated [13,30], most species lack the closed vascular systems required by RTN theory [13,20,36], evidence is accumulating that metabolic scaling is not a simple result of body size-related limits in oxygen and nutrient supply to metabolizing cells [27,29,30,98,146], and RTN theory is incapable of explaining the systematic effects of many kinds of intrinsic biological factors (e.g. body shape and composition, activity level, mode of thermoregulation, growth rate, etc. [13,25,26,28–30,52,53,69,72,73,95]) and extrinsic ecological factors (e.g. temperature, pH, food, predation risk, etc. [13,30,40,56,57,62,69,81,83,85–88,90,96,97]) on *b*.

In addition, several investigators now view physical or geometric constraints as acting as boundary limits on the variation of *b*, rather than as the cause of any central tendency in *b* [8,13,25,42,53,60,64,69,72,82,106,147]. For example, the MLBH posits that the simple geometric properties of surface area and volume may help to explain why *b* often varies between 2/3 and 1 in isomorphic organisms with isometric resource-demanding processes, and over even larger ranges in organisms with variable body shapes or allometric scaling of specific resource-demanding processes [13,25,26,28,30,56,69,87] (see also [15,82]). Accordingly, high frequencies of *b* values in the middle of a frequency distribution for multiple diverse species do not necessarily follow from a single predominant mechanism, as sometimes thought [4,19,32,126], but may instead be a mere statistical result of adaptive variation occurring between two boundary limits (e.g. 2/3 and 1 [25,82]).

**Figure 5.**
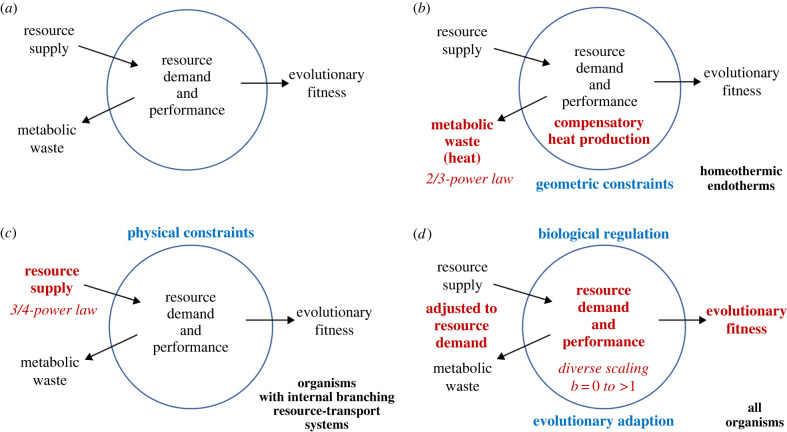
Schematic representations of how metabolic scaling may be affected by physically constrained RS or metabolic waste removal, versus variable RD effects of fitness-related activities. (*a*) Major whole-organism processes affecting the rate of metabolism and its scaling with body mass. (*b*) How 2/3-power metabolic scaling may arise from geometrically constrained fluxes of heat across body surfaces that result in compensatory, metabolically produced heat production to maintain a constant body temperature in homeothermic endotherms [11–13,120,144]. (*c*) How 3/4-power metabolic scaling may arise from body size-dependent, rate-limiting fluxes of resources through internal vascular networks to metabolizing cells in organisms with closed branching vascular systems. (*d*) How diverse metabolic scaling in many kinds of organisms may arise from variable body size-dependent RDs of various biological processes or structures that affect fitness-related organismal performance (including growth, reproduction, locomotion and survival), with co-adjusted effects on RS and metabolic waste removal. (Online version in colour.)

Furthermore, although traditional theory focusing on the surface law or 3/4-power law has emphasized how physical and geometric constraints on RS and (or) waste removal may cause *b* to be 2/3 or 3/4 (figure 5*b,c*), recently many investigators have identified body size-dependent variation in RD by various vital biological structures and processes (e.g. growth, reproduction, locomotion and thermoregulation) as a major cause of variation in *b* (figure 5*d* [8,13,25,29,30,43,46,52,53,57,69,72–74,77,101,129,130,144,146]). A RD-centred view has four major advantages over a RS-centred view of metabolic scaling. First, a simple RS view is contradicted by growing evidence that RS to metabolizing cells is not necessarily body size-related [13,27,29,30,95,146]. Furthermore, modern advances in biochemistry have shown that the rates of various metabolic reactions may be controlled by RD, and not just RS, as traditionally thought (reviewed in [148]). Second, a RD view can more easily explain the effects of various biological and ecological factors on *b* than can a RS view, which involves internal physical and geometric constraints that are presumed to act independently of biological state and various environmental conditions [8,13,30,98]. For example, a recent study of a freshwater amphipod crustacean has shown that isolated spring-dwelling populations exposed to fish predators have significantly lower, remarkably similar *b* values for metabolic rate, growth rate and gill surface area compared to those not exposed to fish predators [149,150]. This parallel allometry is more easily explained as a result of size-selective predation favouring changes in the ontogeny of resource-demanding growth, which in turn alters the ontogenetic scaling of respiratory metabolism and the gill surface area supporting it, rather than predation having an implausible direct effect on oxygen-supplying gill surface area, which in turn affects metabolism and growth [149,150]. In short, predation seems to have altered metabolic scaling more by a direct effect on RD than RS. Third, the performance (survival, growth and reproduction) and ultimately the evolutionary fitness of an organism is more directly related to RD than to RS (figure 5). After all, an organism can more easily control internal RD processes than RS levels in the external environment. Fourth, multiple lines of evidence show that RS within an organism is more a function of RD than the reverse [3,13,20,22,25,43,82,98,151]. For example, increased exercise in animals can significantly alter the anatomy and functioning of vascular RS networks in multiple ways [13,43]. Following a Darwinian worldview, processes related to RS and metabolic waste (including heat) removal may act as extreme boundary constraints on *b*, but within these limits, *b* may normally be a function of the RD of various regulated and adaptively evolved fitness-related activities (figure 5*d*).

Lastly, although ‘Newtonian’ law-based approaches to metabolic scaling theory focus primarily on how organisms physically uptake, transport and use energy and other resources to support metabolism, ‘Darwinian’ context-based approaches additionally focus explicitly on how biological information, as embodied in various genetic and regulatory systems, is used to control the uptake, transport and use of resources (figure 5). Consequently, Darwinian approaches are more complete, as they fully embrace the two major aspects of life: i.e. how it processes both resources and information. I contend that a realistic, comprehensive view of metabolic scaling should recognize organisms not just as resource users, but as ‘informed resource users’ [13]. By doing so, the flexibility and adaptability of the body mass scaling of metabolism and associated biological processes are more easily understood [13,148,150].

## 7. Conclusion and prospects

Based on the extensive evidence discussed in this review, I do not believe that it is too outlandish to say that a scientific revolution in our understanding about metabolic scaling is occurring. In a scientific sense, investigators studying metabolic scaling are increasingly appreciating ‘diversity and inclusion’ by showing more awareness of the empirical diversity of metabolic scaling patterns, as well as more inclusiveness about the kinds of theory used to explain this diversity. For 25 years, RTN theory that has focused primarily on an ideal, non-existent 3/4-power law has dominated the metabolic scaling field, but many investigators are now invoking multiple mechanisms to explain the diversity of metabolic scaling that actually exists [13,29,30,52,53,98,144]. General theory need not be based on a single primary deterministic mechanism, but may include multiple mechanisms that act in a context-dependent way [13,30]. Darwinian approaches to metabolic scaling that embrace multi-mechanistic theory are especially appropriate because the theory of natural selection is itself multi-mechanistic. Indeed, the contingent action of many kinds of genetic and environmental factors is involved in the adaptive evolution of organisms by natural selection in diverse local habitats.

New multi-faceted Darwinian approaches focused on adaptable phenotypic plasticity and evolvability show much promise for increasing an understanding of the diversity of metabolic scaling. Recommendations for further research on little-understood topics include studies examining (i) how biological regulatory systems at the molecular, cellular and organismal levels control the phenotypic plasticity of metabolic scaling [13,27,53,77,90,98,148]; (ii) the quantitative genetic basis for the evolvability of metabolic scaling relationships [38,77], including genetically based estimates of *b* [103]; (iii) relationships between the (co)variation of metabolic rate and body mass and various estimates of evolutionary fitness associated with growth, reproduction and survival [38,53,129,152]; (iv) phylogenetic studies of the evolution of *b* in relation to diverse intrinsic and extrinsic factors [67,104–106]; (v) the mechanisms causing microevolution of *b* within conspecific populations, and macroevolution of *b* across species, including, in particular, multi-variate selection on metabolic rate, body mass and other related traits [38,103,144]; (vi) the effects of various physical, developmental or evolutionary constraints on the boundary limits of *b* [8,25,29,60,69,82,147]; and (vii) interactive effects of RS and RD processes on metabolic scaling, and how they are influenced directly and indirectly by various interactive biological and ecological factors [13,29,86–88,148]. In short, holistic system analyses involving both proximate (functional) and ultimate (evolutionary) causal factors operating at multiple hierarchical levels in many kinds of organisms and environments are required to elucidate fully why metabolic scaling is so diverse [8,13,29,30,53,78,144]. Incisive comparative analyses and controlled experiments, including laboratory and field manipulations of the magnitude of specific RS and RD processes, and artificial selection targeting these specific processes should be especially helpful in determining their relative contribution to metabolic scaling relationships under different conditions [8,29,101,102]. An extraordinarily difficult challenge will be to assess the effects of various factors on the scaling of metabolic rate measured under natural, highly variable field conditions, rather than under artificially controlled conditions in the laboratory, as is usually done [144]. Other useful recommendations for further research can be found in [144].

An increased appreciation of the extensive, law-breaking evidence for variable scaling of metabolism and other associated biological processes has many important practical scientific, medical, agricultural, forestry and conservation implications. For example, comparative studies of variation in metabolic rate or other related traits can no longer control for effects of body size by simply assuming that metabolic rate scales with body mass according to a universal 3/4-power law, but must now consider complex interactions between body size and various other factors of interest, as revealed by significant effects of these factors on *b* [68]. In addition, the demise of the 3/4-power law is revolutionizing medical protocols for administering drug dosages to humans, who exhibit significantly different *b* values for rates of metabolizing drugs based on age and drug type [13,153,154]. The predictions of many theoretical models in ecology, forestry and conservation biology may also be improved by recognizing the diversity of metabolic scaling [13,23,25,102,127,155].

The big picture is no longer a physically constrained, deterministically caused 2/3- or 3/4-power scaling law applying to most or all of life, but rather the pervasive occurrence of extensive variability in metabolic scaling at multiple taxonomic levels, owing to adaptable phenotypic plasticity and genotypic evolution that are highly sensitive to a variety of biological and ecological influences that are best understood with multi-mechanistic, context-dependent theory. In short, metabolic scaling is a ‘many-splendoured thing’ [98, p. 1633].

## Data Availability

This article has no additional data. Publications counted in figure 1 and those used to supply data for figure 2 are listed in the electronic supplementary material. The data are provided in electronic supplementary material [156].
